# Evaluation of the Impact of Parylene C Deposition Method on the Functional Properties of Fabrics

**DOI:** 10.3390/ma17164073

**Published:** 2024-08-16

**Authors:** Pamela Miśkiewicz, Adam K. Puszkarz, Waldemar Machnowski, Andrzej Nosal

**Affiliations:** 1 Institute of Architecture of Textiles, Faculty of Material Technologies and Textile Design, Lodz University of Technology, 116 Żeromskiego Street, 90-924 Lodz, Poland; 2 Textile Institute, Faculty of Material Technologies and Textile Design, Lodz University of Technology, 116 Żeromskiego Street, 90-924 Lodz, Poland; waldemar.machnowski@p.lodz.pl; 3 Institute of Materials Science and Engineering, Faculty of Mechanical Engineering, Lodz University of Technology, 1/15 Stefanowskiego Street, 90-924 Lodz, Poland; andrzej.nosal@p.lodz.pl

**Keywords:** Parylene C, coating, fabric, contact angle, air permeability, thermal insulation, charge decay, flammability, micro-CT

## Abstract

The article presents the results of research on the impact of the use of an original, innovative method of deposition of Parylene C on the functional properties of fabrics with various potential applications (e.g., thermal and chemical protective clothing, packaging, covers and others). Verification of the effects of the method used was based on interdisciplinary research taking into account the impact of coating fabrics on changes in their structure (micro-CT), surface properties (contact angle), barrier properties (water and chemical liquid wetting), electrostatic properties (charge decay), biophysical properties describing heat and mass transfer (by the Alambeta system and thermal imaging) and flammable properties. Four fabrics made of synthetic organic fibres (meta-aramid, para-aramid) and natural inorganic fibres (basalt) were selected for testing. Given the complex structure of textile substrates, the results confirmed that the two assumed thicknesses of the Parylene C coating were consistent with the actual measurements. The findings indicated that the coatings significantly reduced water and acid absorption in the fabrics compared to unmodified ones. Thermal insulation property tests revealed that coated fabrics exhibited higher thermal conductivity than unmodified fabrics. Additionally, the presence of Parylene C on aramid fabrics resulted in a modest increase in their ignition resistance.

## 1. Introduction

Parylene, also known as a polymer, based on poly-para-xylylene, is a crystal clear, amorphous and polycrystalline linear polymer. The Parylene coating is smooth and transparent. Moreover, it can perfectly adapt to the ground because it has good adhesion [1,2,3].

**Figure 1:**
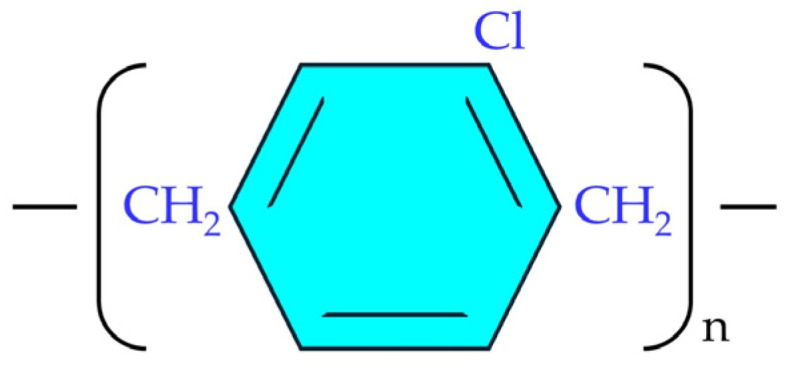
The monomer of Parylene C.

There are over 10 different commercial varieties of Parylene in the world. The basic variety is Parylene N (p-xylylene). It has the best ability to penetrate holes and gaps and has the highest dielectric strength. Its disadvantages include the longest deposition time. The next one is Parylene C (poly[chloro-p-xylylene]), the most widely used of all Parylene varieties [3,4]. One chlorine atom is substituted on the aromatic ring of Parylene C (Figure 1).

Moreover, it is an organic coating without any plasticizers, catalysts and solvents [5]. It has a number of features used in medicine, such as low water absorption, low coefficient of friction and high breaking elongation. Parylene D (poly[dichloro-p-xylylene]) has comparable properties to Parylene C, and additionally, it is characterized by the highest thermal stability [4]. The deposited layers are hard coatings with low elasticity, and the compound itself has the worst ability to penetrate the material surface. Due to this, Parylene D is most often used in industrial applications where strength, chemical and thermal resistance are important [3,4,5]. Parylene HT (poly[tetrafluoro-p xylylene]) is also becoming more and more popular. This is due to its extraordinary ability to penetrate cracks, higher thermal stability, lower dielectric constant, minimal moisture absorption and higher resistance to ultraviolet rays [6,7].

Parylene coatings, thanks to their multi-molecular carbon structure, are characterized by excellent resistive properties and high dielectric resistance [8,9]. Another advantage of the coatings is their tear strength and high elasticity. Their relatively low level of friction and lack of internal stresses make them often used in medicine—especially as laminates on prostheses and pacemakers in biomedicine [10]. Coatings also have hydrophobic properties—water molecules are unable to penetrate them. Moreover, they constitute an excellent barrier against penetrating particles of various substances, e.g., gases, due to their low permeability [5]. Moreover, it is resistant to most acids, salts, and alkalis.

Currently, one of the main applications of Parylene coatings is their use in electrical circuits as protection against corrosion resulting from extreme weather conditions. The parylene coating does not interfere in any way with the operation of the electrical system, and due to its high ability to penetrate, it is mainly used for micro circuits with a diameter of up to one millimetre [11,12].

Parylene coatings present their full potential in biomedicine, where they are used in implants, pacemakers and prostheses, due to its biocompatibility, low level of friction and permeability and crystalline structure [13]. The role of Parylene in implants placed inside the human body is significant because it can block the entry of unwanted and toxic metal ions, such as iron, chromium or nickel, into the human body [6]. However, in dentures, Parylene is used as a coating for rubber parts [13,14].

Another application of the Parylene coating is the preservation of natural elements or of animal and plant origin [15]. In this case, the ability of the coating to be deposited on the entire surface of the sample and the ability to penetrate every crack or capillary is used.

Despite its numerous attractive functional properties, Parylene C has not been studied as far as a material for coating textiles in order to provide them with various utility properties (such as hydrophobicity, resistance to wetting, resistance to penetration by water and liquid chemical substances, electrostatic, thermal insulation, flammability and others). A probable reason for this may be the fact that textile substrates characterized by a complicated, porous fibre system make it exceedingly difficult to obtain uniform and continuous layers on them. One of the main questions asked by the authors of the paper was whether it is possible to depose Parylene C using the CVD process resulting in noticeable changes in the functional properties of the modified fabrics. The question is justified for at least two reasons. One of them is the specificity of the deposition process itself (related to, among others, the selection of appropriate thermal, pressure and geometric conditions). The second reason influencing the effectiveness of the applied method is the selected substrates, characterized by a complicated spatial geometry, which significantly hinders the possibility of obtaining continuous uniform coatings on them.

The authors of the current article attempted to use a Parylene C coating in composites with potential use in gloves protecting against hot work environments. The role of the Parylene C coating was to improve the resistance to contact heat and radiant heat of the tested composites, as well as to improve the sensory comfort of the inner layer of the composite (lining) made of basalt fabric [16].

The aim of this article was to assess the impact of the original Parylene C coating method on selected fabric properties. Fabrics made of synthetic organic fibres (meta-aramid, para-aramid) and natural inorganic fibres (basalt) were selected for modification. The modified fabrics were evaluated for various, often unrelated, functional properties. Using ten different verification methods, the authors verified the impact of the Parylene C deposition method on the functional properties of fabrics related to their use in environments that may have a positive or negative impact on the safety of their user. Wide selection of functional properties was intended to show users of various industrial sectors the benefits and limitations of using Parylene C and the specific method of modifying selected textile substrates with it. The conducted experiments examined, among other aspects, how textile modification affects their:

- *surface properties* (especially hydrophobicity), which may be important when using textiles in special clothing (e.g., jackets, coats, uniforms for firefighters, rescue), special equipment (e.g., backpacks, tarpaulins), packaging used in high-humidity conditions (e.g., during transport).
- *barrier properties* protecting against the harmful effects of liquid chemicals (e.g., acids and bases), which may be important when using textiles in protective clothing or packaging used in places where these substances occur (e.g., chemical or medical laboratories).
- *electrostatic properties*, which is important when using these textiles in areas where there is a risk of explosion by means of uncontrolled charge transfer in the form of a spark (e.g., gas stations, mills, sawmills, mines and others).
- *properties affecting heat and mass transfer through them*, affecting the use of textiles as a barrier between environments with different temperatures and pressures. When used in clothing and footwear, these properties have a significant impact on ensuring the heat balance between a person and the environment in which he or she lives and protecting him or her from hypothermia (in cold environments) or from overheating (in hot environments). In other applications (e.g., packaging), these properties may contribute to ensuring the thermal safety of packaged products.
- *flammability properties* that may be important for textiles that may come into direct contact with flame (e.g., firefighting, rescue and clothing and equipment for metallurgical industry).

The use of high-resolution X-ray computed tomography (micro-CT) was aimed at examining and describing the impact of changes in the microstructure of fabrics caused by the applied deposition of Parylene C on changes in their functional properties mentioned above.

## 2. Materials and Methods

### 2.1. Materials

**Figure 2:**
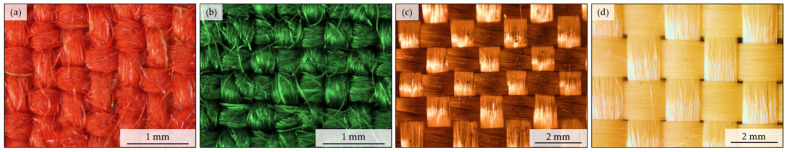
The optical microscopy images of tested fabrics: red (**a**), green (**b**), orange (**c**), yellow (**d**).

The subject of the research was comprised of four fabrics: meta-aramid with a hydrophobic finish (red), meta-aramid (green), basalt (orange) and para-aramid (yellow). In addition to their raw material composition, fabrics vary in terms of thickness, mass per unit area, weft and warp density and porosity. All fabrics except the red fabric (twill) have the same weave (plain). The physical parameters of the fabrics are presented in Table 1, while Figure 2 shows their photos taken using an optical microscope Delta Optical Smart 5MP PRO (Delta Optical, Warsaw, Poland) equipped with firmware: Delta Optical Smart Analysis Pro 1.0.0.

As can be seen in the optical microscopy images, the green and red fabrics are meta-aramid staple fibre-based fabrics (Figure 2a,b), while the orange fabric (Figure 2c) and yellow fabric (Figure 2d) are made of continuous fibre yarn (basalt and para-aramid, respectively).

The surface of both fabrics made of filament yarns is smooth and lustrous, while in the case of red fabric and green fabric, there are numerous fibres separated from the yarns and protruding above their surface.

### 2.2. Methods

#### 2.2.1. Parylene C Coating Method

In the Parylene C deposition technology developed in the 1960s by Gorham [1,19], the unique polymerization mechanism of chloro-para-xylylene consists in the lack of the participation of an external reaction initiator. In this case, the polymerization reaction is initiated by a monomer molecule in the excited state, and the chain propagation reaction of poly(chloro-para-xylylene) with the trade name Parylene C is of a free radical nature [20]. This mechanism of building polymer chains results in their extraordinary purity compared to standardly produced polymers. The technology also has another unusual property, namely that the monomers produced in it are in the gas phase during the pyrolysis of di(chloro-para-xylylene)—Parylene C dimer and in this state, they are delivered through a diffuser that disperses a stream of monomers to the surface of the samples on which the polymerization reaction takes place at room temperature. The most important advantages of this process resulting from the diffusion mechanism of delivering monomers to the surface are conformity [3] and extremely effective penetrating abilities [21] allowing penetration of exceedingly small spaces, building a coating regardless of the complexity of the surface topography. Thanks to these properties, unique in other polymer technologies, the Parylene C coating is ideal for covering textile products.

**Figure 3:**
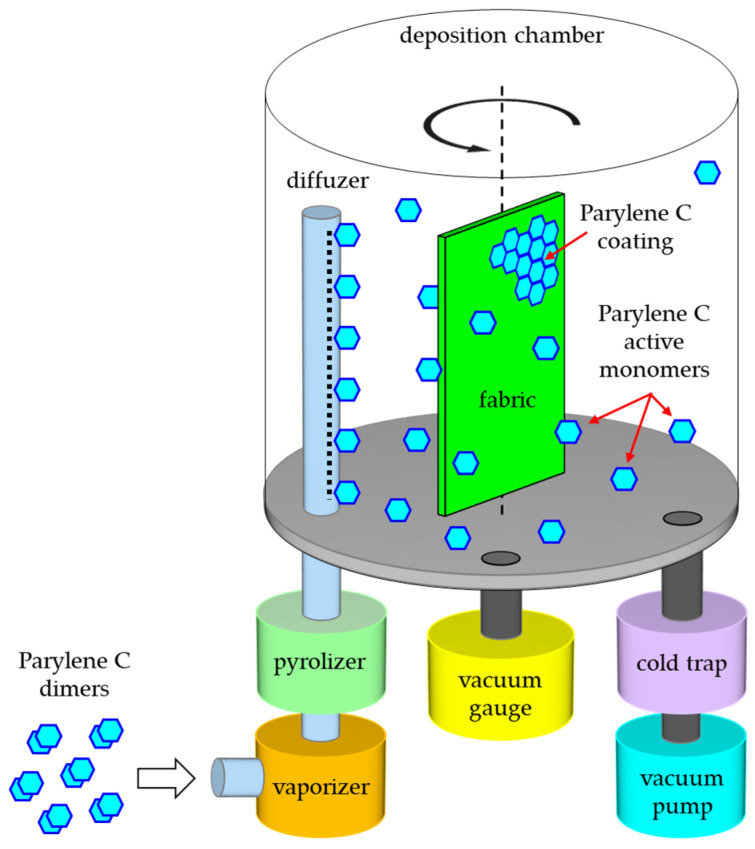
Th scheme of applied method of coating fabrics with Parylene C.

Commercial equipment from Specialty Coating System (Indianapolis, IN, USA) was used to cover fabrics with Parylene C, the scheme of which is shown in Figure 3. The fabric subjected to deposition is placed on a holder that allows the fabric to be stretched flat. The prepared holder is placed on the rotary table in the deposition chamber. The maximum size of the modified fabric did not exceed the surface area of an A4 sheet. The sublimator contains the appropriate amount of Parylene C for a given deposition process. After tightly closing the vacuum system and pumping down to the maximum pressure (not exceeding a dozen or so Pascals), when the pressure stops dropping and reaches a value below the base pressure we assumed, the cold trap is flooded with liquid nitrogen to protect the vacuum pump from being covered with a Parylene coating from the inside. The pressure in the process chamber, which regulates the rate of coating deposition, is controlled by a vacuum gauge. After turning on the table’s rotating mechanism, the vacuum probe and pyrolizer are heated. When the pyrolizer temperature approaches the set 690 °C, the sublimator is automatically heated and its temperature increases until the assumed deposition pressure is reached. This value increases from room temperature to a maximum of 175 °C as the dimer decreases. If, despite the increase in temperature, the deposition pressure begins to decrease, the process is considered completed. Then, all heating elements are automatically turned off and after they cool down to room temperature, the process chamber is ventilated, and the coated fabrics are ready for use.

Two different thicknesses (15 μm and 30 μm) of Parylene C coating were deposited to each fabric in order to examine the real amount of polymer coating, its uniformity and influence on some important properties of the modified fabrics.

#### 2.2.2. The Influence of Coating on Structural Properties of Fabrics

##### X-ray Computed Tomography, Micro-CT

High-resolution X-ray computed tomography, micro-CT (SkyScan 1272; Bruker, Kontich, Belgium) was used to investigate the effect of Parylene C coating on the structural parameters of fabrics. The fabrics were scanned under the following conditions: X-ray source voltage: 50 kV, X-ray source current: 200 µA, pixel size: 5 µm, rotation: 180° rotation, rotation step: 0.2°, without filter. Using the software NRecon 1.7.4.2, CTAn 1.17.7.2+ and Data Viewer 1.5.6.2 software (made by Bruker), the following fabric parameters were determined: (1) total porosity, (2) yarn porosity, (3) thickness distribution of the Parylene coating on the fabric surface. Using the CTvox 3.3.0 r1403 software (made by Bruker), three-dimensional visualizations of fabrics with the identified Parylene C coating were made.

#### 2.2.3. The Influence of Coating on the Wettability and Tightness of Fabrics

To investigate the impact of the applied fabric coating method on their surface wetting by water and tightness to chemical liquids and air, the four techniques were selected.

##### Contact Angle

**Figure 4:**
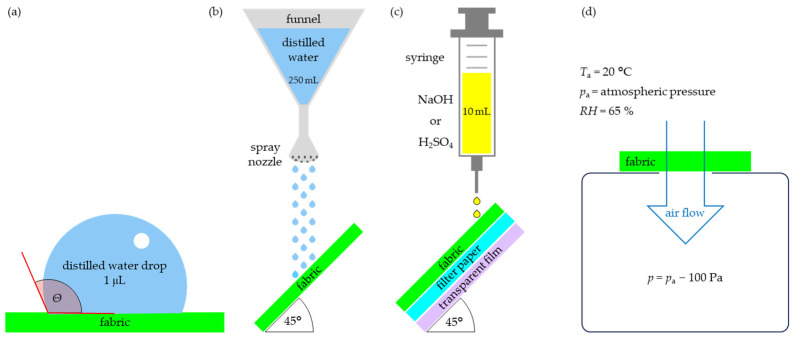
Th schemes of the measurement methods used to assess the impact of coating on the wettability and tightness of fabrics: (**a**) contact angle, (**b**) spray test, (**c**) chemical liquids penetration, (**d**) air permeability.

Firstly, it was examined how the Parylene C coating changes the wetting properties of fabrics by measuring the *contact angle, Θ*. In this method, drops of distilled water with a volume of 1 µL were placed on the surfaces of unmodified fabrics and fabrics with a Parylene C coating. The contact angle measurement system consisted of an optical system power supply from OPTEC s.c. (Białystok, Poland) and a digital camera, model Infinity 1-1c by Teledyne Lumenera (Ottawa, ON, Canada). Contact angle measurements were made using DROP software (version 2.1, 2005) by Stanisław Sosnowski (Polish Academy of Sciences, Lodz, Poland). The scheme of the method is shown in Figure 4a.

##### Spray Test

Secondly, to determine resistance to surface wetting of unmodified and coated fabrics, the coatings were subjected to the next method—*spray test* according to *EN ISO 4920* [22]. A 250 mL volume of distilled water was sprayed on a test fabric that has been mounted on a ring and placed at an angle of 45° so that the centre of the specimen was at a specified distance below the spray nozzle. The scheme of this method is presented in Figure 4b. Depending on the properties of the sample surface, water soaks into the fabric or flows down its surface without wetting it. The spray rating was determined by comparing the appearance of the specimen with the photographic spray rating ISO standard scale. A six-level ISO rating scale is used: *ISO 5* means the highest resistance to surface wetting (“no sticking or wetting of the specimen”); *ISO 0* means the lowest wetting resistance (“complete wetting of the entire face of the specimen”).

When using this method, the percentage weight increase of the samples (Δ*m*) was additionally determined due to the water absorption.

##### Chemical Liquids Penetration Test

The test method described in EN ISO 6530:2005 [23] for resistance of unmodified and coated fabrics to penetration by liquid chemicals was used. The scheme of this method is presented in Figure 4c. Sulfuric acid (30%) and sodium hydroxide (30%) were used as test liquids. The index of penetration (*p*_H2SO4_ and *p*_NaOH_), i.e., the ratio, expressed as a percentage, of the amount of liquid that accumulated in the filter paper to the original amount of liquid poured onto this fabric, was determined for all tested fabrics.

##### Air Permeability

Finally, the effect of Parylene C coating on the air permeability of the tested fabrics was determined using the method according to PN-EN ISO 9237:1995 [24] using an air permeability tester (FX 3300, Textest Instruments, Schwerzenbach, Switzerland). The scheme of the experiment is presented in Figure 4d.

#### 2.2.4. The Influence of Coating on Electrostatic Properties of Fabrics

##### Charge Decay, Surface Resistance and Vertical Resistance

**Figure 5:**
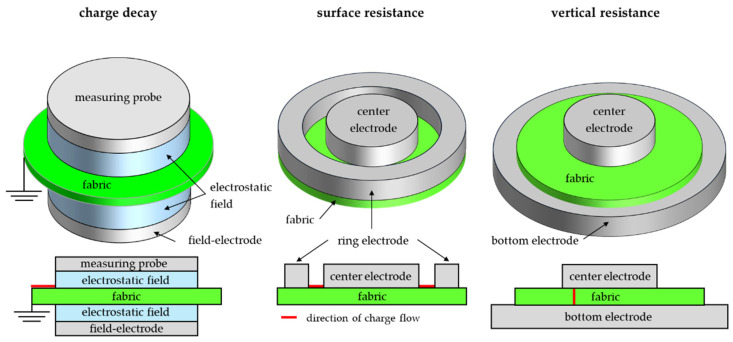
The schemes of measurement systems of electrostatic properties of tested fabrics.

To investigate how deposition of Parylene C coatings affects the electrostatic properties of the fabric, measurements of charge decay were performed according to PN-EN ISO 1149-3 [25]; as well, surface resistance and vertical resistance were carried out according to PN-EN ISO 1149-1 [26] and PN-EN ISO 1149-2 [27], respectively. Schemes of the techniques used are shown in Figure 5.

#### 2.2.5. The Influence of Coating on Thermal Insulation Properties of Fabrics

##### Thermal Conductivity Coefficient

**Figure 6:**
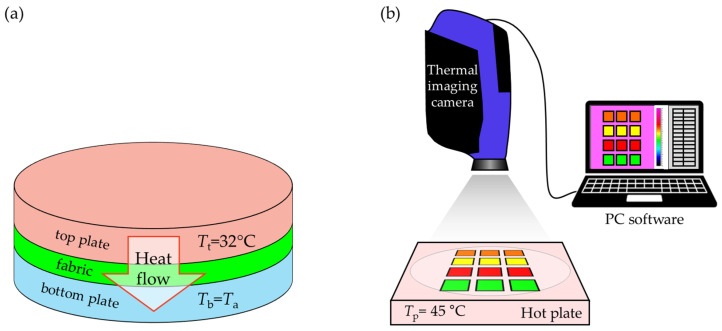
The schemes of the Alambeta system (**a**) and thermal imaging (**b**).

To investigate the influence of the Parylene C coating deposition method on the thermal conductivity coefficient of fabrics, an Alambeta device was used (Sensora, Liberec, Czech Republic). The measurement of thermal conductivity is based on the measurement of the heat flux generated by a temperature difference between the top plate of the device of 32 °C, and the bottom one at ambient temperature. The heat passes from the top plate to the bottom plate through the fabric placed between the plates. A scheme of the measurement method is shown in Figure 6a.

##### Thermal Imaging

Knowing the thermal conductivity coefficient of fabrics obtained by measurements using the Alambeta system, an additional research technique using thermal imaging was used to understand the impact of the Parylene C deposition method more precisely concerning the heat transfer through fabrics. This technique allowed for a more detailed analysis of the process of gradual change in fabric temperature due to the heat supply. The experiment scheme is shown in Figure 6b. The fabrics were placed on a hot plate (e-G51HP07C Guardian 5000 model, OHAUS Europe GmbH, Nänikon, Switzerland), the temperature of which increased over time from the ambient temperature of 25 °C to 45 °C. The temperature difference between the hot plate and the surroundings, ranging from 0 to 20 °C, resulted in heat transfer (mainly vertical) from the heating plate to the surroundings through the fabrics. A thermal imaging camera (FLIR SC 5000 model made in Wilsonville, OR, USA) placed above the plate and attached software (Altair by FLIR Systems, version.5.90 002, 2010) recorded the increasing temperature of the top surface of the fabrics. Thermal imaging was conducted until the fabrics reached a steady state, when the temperature of the top surface of the fabric *T*_top_ reached the maximum possible and constant value in time. The following parameters were determined based on the dependence of the fabric temperature on time: (1) *heating rate* and (2) *maximum temperature* reached by the fabrics in the process of heating up.

#### 2.2.6. The Influence of Coating on Flammability of Fabrics

##### Limiting Oxygen Index

The limiting oxygen index (*LOI*) is the method most frequently used to examine the improvements or deterioration in flame retardancy of materials because of subjecting them to various processes of modification. Therefore, the *LOI* method was used to test the flammability of unmodified fabric samples and fabrics coated with Parylene C. *LOI* values for fabrics samples were determined according to EN ISO 4589-2 [28] using an Oxygen Index Apparatus (Fire Testing Technology, Charlwoods Road, East Grinstead, West Sussex, RH19 2HL, UK). The higher the *LOI* value of a material, the greater its resistance to ignition.

## 3. Results and Discussion

### 3.1. The Influence of Coating on Structural Properties of Fabrics

High-resolution X-ray microtomography (micro-CT) was used to investigate the effect of Parylene C deposition on the structure of fabrics. First, it was checked whether both assumed deposition thicknesses of Parylene C coatings (15 μm and 30 μm) were achieved on all fabrics. Due to the difference in the absorption of X-rays of the coating and the fabric, it was possible to identify and statistically quantitatively analyse the Parylene C in fabrics subjected to deposition.

**Figure 7:**
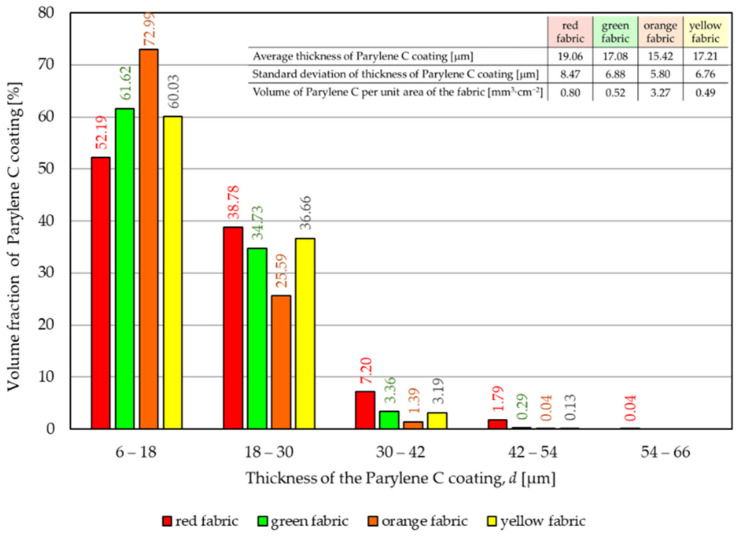
The distribution of the thickness *d* of the Parylene C coating obtained for the deposition in which it was intended to achieve a 15 μm layer.

Figure 7 shows the distribution of the thickness *d* of the Parylene C coating obtained for the deposition in which it was intended to achieve a 15 μm layer on four tested fabrics.

Based on the distribution, it can be observed that in all modified fabrics, the largest part of the Parylene C coating has a thickness *d* in the range of 6 μm–18 μm. In the orange fabric, this part constitutes 72.99% of the total Parylene C content in the fabric, while in the red fabric it is 52.19%. For all fabrics, the second largest portion of the Parylene C coating (ranging from 25.59% for the orange fabric to 38.78% for the red fabric) has a thickness ranging from 18 μm to 30 μm. The remaining content of Parylene C coating in fabrics is in the range of 30 μm–66 μm. Based on the calculated average of Parylene C thickness distribution (listed in the table in the upper right corner of Figure 7), it follows that the coating thickness closest to the intended one (15 μm) was obtained for the orange fabric (15.42 μm), and the least similar for the red fabric (19.06 μm). However, in the case of the deposition of a 15 μm Parylene C coating, the maximum difference between the assumed and real coating thicknesses is 27% for the red fabric, while the minimum difference is 3% for the orange fabric. As part of the structural analysis, the volume of Parylene C per unit area of fabric was also calculated, which ranged from 0.49 mm^3^·cm^−2^ to 3.27 mm^3^·cm^−2^ depending on the fabric.

**Figure 8:**
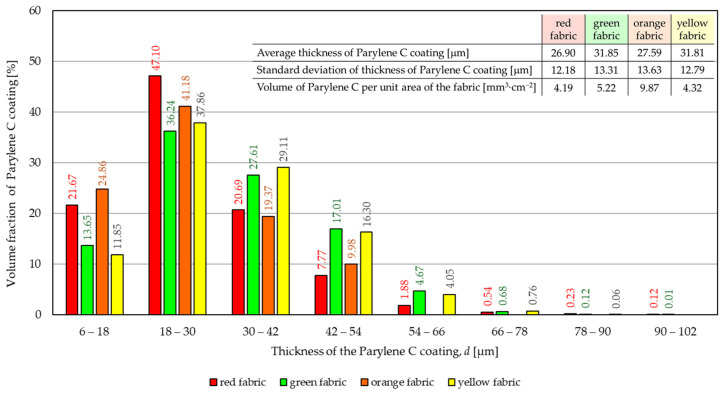
The distribution of the thickness *d* of the Parylene C coating obtained for the deposition in which it was intended to achieve a 30 μm layer.

Figure 8 shows the distribution of the thickness *d* of the Parylene C layer obtained for the deposition in which it was intended to achieve a 30 μm layer on four tested fabrics.

Based on the distribution analysis, it can be observed that in all modified fabrics, the largest part of the Parylene C coating has a thickness *d* in the range of 18 μm–30 μm. In the red fabric, this part constitutes 47.10% of the total Parylene C content in the fabric, while in the green fabric it is 36.24%. For red and orange fabric, the second largest portion of the Parylene C coating (21.67% for the red fabric and 24.86% for the orange fabric) has a thickness ranging from 6 μm to 18 μm, while for green and yellow fabric, the second largest portion of the Parylene C coating (27.61% for the green fabric and 29.11% for the yellow fabric) has a thickness ranging from 30 μm to 42 μm A significant content of Parylene C coating in fabrics is in the range of 42 μm–54 μm (from 7.77% for the red fabric to 17.01% for the green fabric). On the basis of calculated average of Parylene C thickness distribution (listed in the table in the upper right corner of Figure 8), it follows that the coating thickness closest to the intended one (30 μm) was obtained for the green fabric and yellow fabric (31.85 μm and 31.81 μm, respectively), and the least similar for the red fabric (26.90 μm). However, in the case of the deposition of a 30 μm Parylene C coating, the maximum difference between the assumed and actual coating thickness is 10% for orange fabric and 6% for green and yellow fabric. As part of the structural analysis, the volume of Parylene C per unit area of fabric was also calculated, which ranged from 4.19 mm^3^·cm^−2^ to 9.87 mm^3^·cm^−2^ depending on the fabric.

Observing the shape of the Parylene C coating above distributions for two assumed coating thicknesses (15 μm and 30 μm) of the deposited polymer, it should be noted that for a coating twice as thick, the distribution is wider by 3 classes covering the coating thickness range from 66 μm to 102 μm. The shape of both distributions may lead to the conclusion that it is not an easy task to obtain a coating of the desired thickness, evenly distributed on textiles, in the applied deposition process. Fabric is a complex spatial arrangement of fibres connected in yarns and therefore a much more demanding object for surface modification in comparison to smooth substrates such as glass, wall, sheet metal, etc., which was also confirmed in similar approaches [29,30]. Moreover, it is a system that, in the process conducted in variable pressure and thermal conditions, may be deformed and prevent the deposition process from proceeding precisely. In the case of modification of the abovementioned objects with a flat surface, it is much easier to obtain a uniform coating.

**Figure 9:**
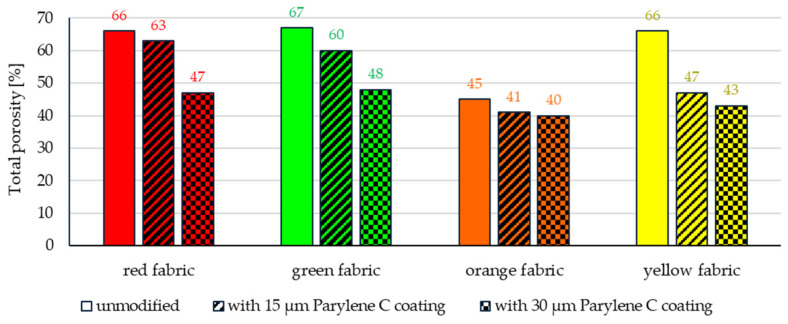
The total porosity of unmodified and Parylene C-coated fabrics.

**Figure 10:**
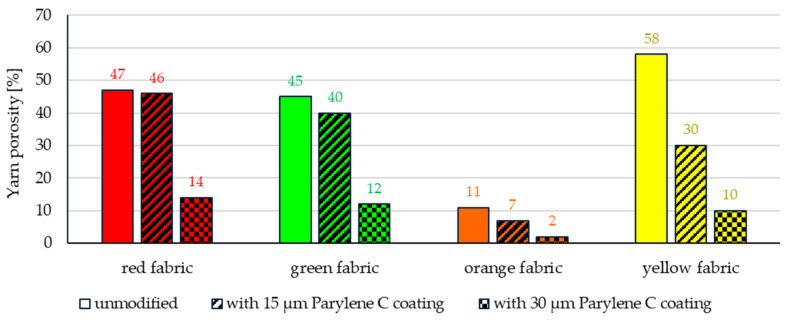
The yarn porosity of unmodified and Parylene C-coated fabrics.

X-ray microtomography for all fabrics also showed the effect of Parylene C coating on changes in total porosity (Figure 9) and yarn porosity (Figure 10).

Both deposition processes (15 µm and 30 µm) reduced the total porosity and yarn porosity of all fabrics, with the deposition of the 30 µm coating causing a much greater change in both porosities. The largest decrease in total porosity was observed for the deposition of 30 µm of the coating in the case of red (from 66% to 47%), green (from 67% to 48%) and yellow fabrics (from 66% to 43%), while the smallest decrease was observed for orange fabric (from 45% to 40%).

As in the case of total porosity, the largest decrease in yarn porosity was observed for the deposition of 30 μm of the coating in the case of red (from 47% to 14%), green (from 45% to 12%) and yellow fabrics (from 58% to 10%), while the smallest decrease was noticed for orange fabric (from 11% to 2%).

The effect of reducing the total porosity and yarn porosity of all fabrics is due to two factors related to the applied method of deposition of Parylene C coatings on textiles. The first is the penetration of the volatile form of Parylene C and its deposition in the free spaces between the fibres inside the fabric yarns. The second one is related to the conditions in the process chamber during deposition. Before the deposition of the polymer onto the fabrics begins, air is pumped out from the process chamber in which the fabrics are located. In the process of lowering the pressure from initial atmospheric pressure, the compression efficiency of fabrics depends on the porosity of unmodified textiles (the higher the porosity, the greater the possibility of reducing the volume of the yarn) and the friction forces between adjacent fibres in the yarn (the greater the loss, the lower the mobility of the fibres towards reducing the volume of the yarn). The friction force between fibres is influenced by the shape of the cross-section of fibres forming the yarn and their spatial orientation in the yarn. When the pressure in the process chamber is reduced, straight fibres arranged parallel to each other in filament yarns have greater mobility than twisted staple fibres in staple fibre-based yarns. In the case of the orange fabric, made of a low-porous (11%) basalt yarn, reducing the pressure in the chamber resulted in low yarn compression, compared to the yellow fabric, made of a much more porous (58%) para-aramid yarn with straight, parallel-arranged fibres.

In the case of the red fabric and the green fabric, the decrease in yarn porosity was less influenced by compression due to the pressure reducing in the chamber, because both fabrics had a more compact geometric structure (with higher warp and weft density). In addition, the yarn in both fabrics had a non-zero twist, which made it much stiffer and less susceptible to deformation. The significant decrease in yarn porosity in the red and green fabrics due to the deposition of the 30 um Parylene C coating was probably caused primarily by the filling of free spaces between the fibres of the yarn by the deposited polymer.

**Figure 11:**
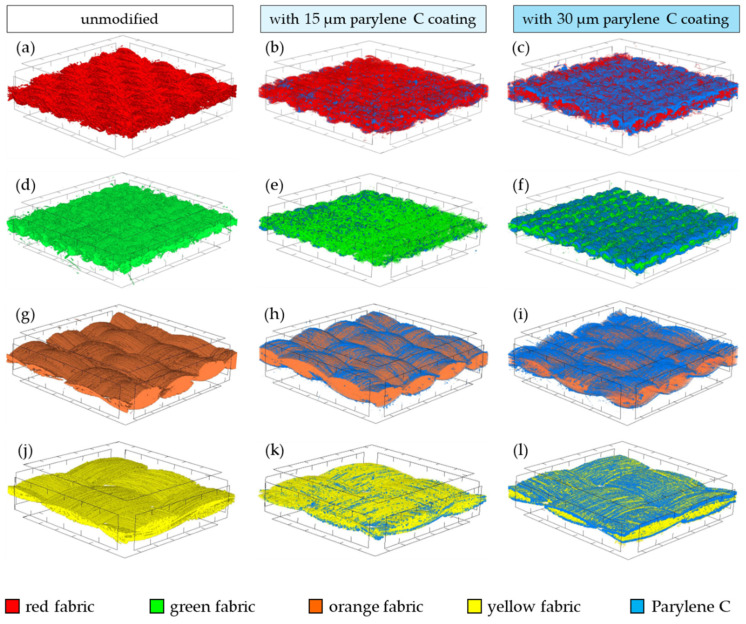
The micro-CT 3D visualization of unmodified and coated fabric: red fabric (**a**–**c**); green fabric (**d**–**f**); orange fabric (**g**–**i**); yellow fabric (**j**–**l**) (all visualizations show a 3 mm × 3 mm fragment of fabric).

Figure 11 shows three-dimensional visualizations of unmodified and Parylene C-coated fabrics obtained using high-resolution X-ray microtomography (micro-CT). All visualizations show a fragment of fabric measuring 3 mm × 3 mm. Thanks to the difference in the X-ray absorption of the coating and the fabric, it was possible to identify and visualize Parylene C (marked in blue).

Based on the visualization, both depositions (15 µm and 30 µm) influenced the different distribution of Parylene C in different fabrics. In the case of the deposition of a 15 µm coating on red, green and yellow fabric, larger amounts of Parylene C penetrated deeper into these fabrics (Figure 11b,e,k) compared to the orange fabric, where most of the Parylene C was deposited on the fabric surface (Figure 11h). The observed significant difference in the spatial distribution of Parylene C depending on the fabric is probably caused by the difference in the yarn porosity of unmodified fabrics (Table 1). Parylene C deposited on fabrics with a much higher yarn porosity (red: 47%, green: 45%, yellow: 58%) compared to orange fabric (11%) could fill the spaces between the yarn fibres to a greater extent. In the case of the orange fabric, Parylene C forms compact, larger coating fragments on the surface of the fabric, while on the surface of the other three fabrics, Parylene C forms a fragmented coating in the form of small islands. In the case of the deposition of a 30 µm coating, the distribution on the surface of all fabrics is similar, where Parylene C forms a thicker and more compact coating.

### 3.2. The Influence of Coating on the Wettability and Tightness of Fabrics

#### 3.2.1. Contact Angle

**Figure 12:**
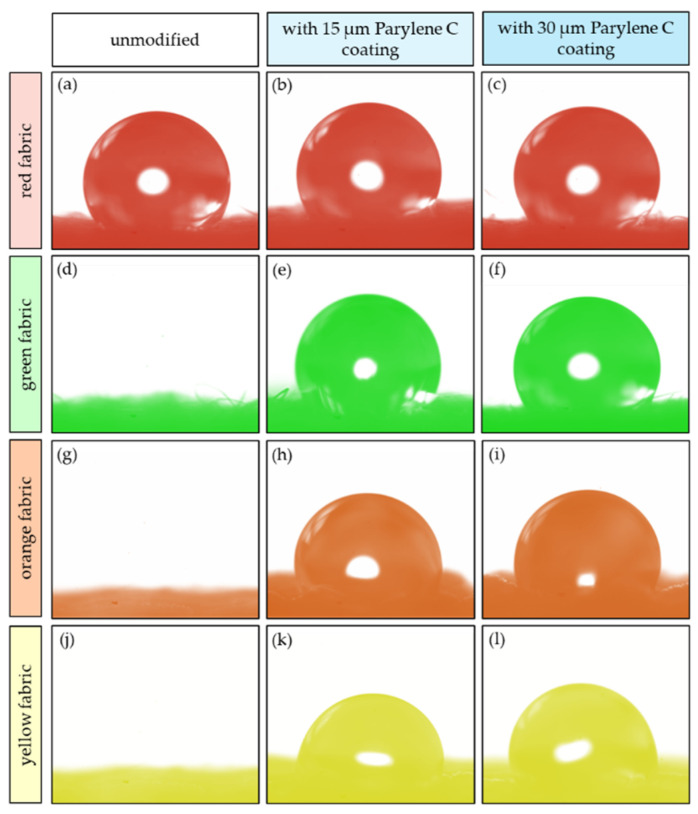
The water drops that were used to measure the contact angle of the unmodified and Parylene C-coated fabric; red fabric (**a**–**c**); green fabric (**d**–**f**); orange fabric (**g**–**i**); yellow fabric (**j**–**l**).

Figure 12 shows photographs of sample water drops that were used to measure the contact angle of the unmodified and Parylene C-coated fabric. The drops and fabrics have been intentionally coloured for better readability and to match the colour scheme used in all charts in the article.

**Figure 13:**
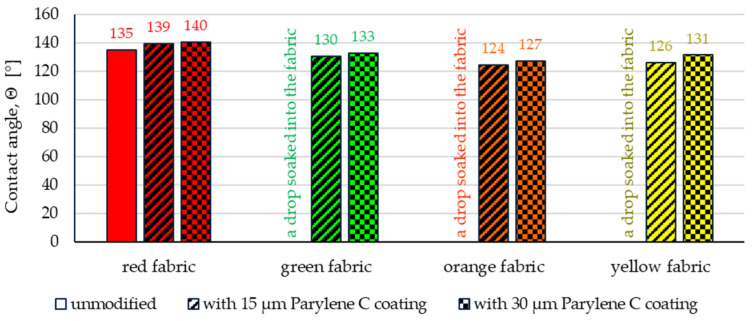
The contact angle of unmodified and coated fabric.

As you can see in the photos, only in the case of the red fabric (water-repellent finish) was it possible to place a stable drop that did not spread out. The results of the contact angle measurements are presented in Figure 13.

Both depositions of Parylene C contribute to the increase in hydrophobicity of all fabrics, while in the case of the green, orange and yellow fabrics, already the deposition of 15 µm coating allows for the placement of a stable drop. On the other hand, the deposition of 30 µm coating contributes to a slight increase in the contact angle for all fabrics: 0.81% (red fabric), 1.74% (green fabric), 2.24% (orange fabric) and 4.22% (yellow fabric). The greater increase in the contact angle between the deposition of 15 µm coating and the deposition of 30 µm coating for orange and yellow fabrics compared to red and green fabrics is probably due to the fact that in the case of basalt and para-aramid fabrics, the deposition of 30 µm coating resulted in a thicker Parylene C coating on the fabric surface. The parameters that could also influence this difference are the geometric parameters of the fabrics (weave, warp and weft density, and porosity). In the case of all tested fabrics, the modification process with Parylene C resulted in an increase in their hydrophobic properties. It should be emphasized that the fabrics’ surfaces, which had excellent wettability before modification (contact angle, *Θ* = 0°), also became highly resistant to surface wetting (contact angle, *Θ* > 124°) after applying a layer of Parylene C. It can therefore be concluded that the Parylene C deposition process used in this study is an effective method of hydrophobizing the fabrics’ surfaces, including fabrics with a high degree of water wettability.

#### 3.2.2. Spray Test

**Figure 14:**
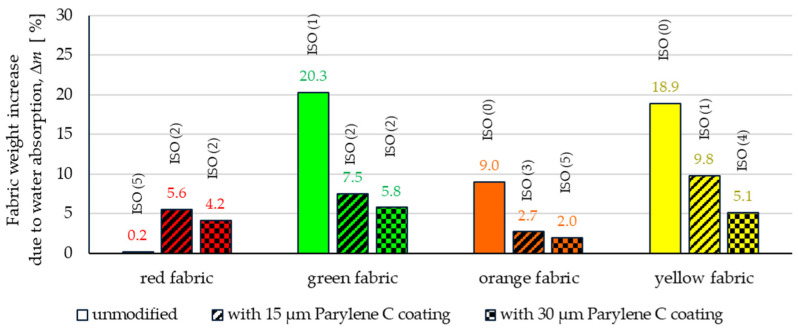
The increase in the weight of unmodified and coated fabrics for the spray test method.

Figure 14 shows the results of measurements of the percentage increase in the weight due to water absorption (Δ*m*) of unmodified and coated fabrics, which were performed during determination of resistance to surface wetting (spray test) according to [22].

Based on the results, it can be observed that, except for the hydrophobically finished red fabric, both depositions (15 µm and 30 µm) of Parylene C coatings result in a significantly smaller increase in the weight of fabrics with coatings compared to their unmodified counterparts. In the case of green and orange fabrics, the greatest decrease in Δ*m* was observed for textiles with a 30 μm deposited coating (the decrease in Δ*m* compared to the unmodified textiles was 14.5% for green fabric and 7.0% for orange fabric). In the case of yellow fabric, the greatest decrease in Δ*m* was observed for the fabric with a deposited 30 μm coating (13.8%). It should be noted that in the case of three fabrics (green, orange and yellow), the differences in Δ*m* between fabrics with a thinner and thicker Parylene C coating are not as significant as in comparison to unmodified fabrics. In the case of the red fabric, both Parylene C depositions increased the fabrics’ ability to absorb water. The deposition of a thinner coating resulted in an increase in Δ*m* by 4.0%, while the deposition of a thicker coating resulted in an increase in Δ*m* by 5.3%. The difference in the impact of deposition on the hydrophobic properties of the red fabric and the other three textiles can be attributed to several reasons. First, the red fabric was the only fabric with a hydrophobic finish among all the tested textiles. Another surface modification, the deposition of Parylene C, could eliminate the effect of surface hydrophobicity by partially filling the space between the fibres inside the yarn, creating tight “micro-pockets” for water that got into them during the experiment. The fact that the red fabric has a different weave (twill) from the other tested textiles (plain) may also be important.

In the bar graph presented in Figure 14, the value of the six-point ISO scale, which of the standard describing the spray test method determines the degree of wetting of the fabric surface, is shown above the Δ*m* bars. According to this ISO scale, *0* means *complete wetting of the entire surface fabric*, while *5* means *no sticking or wetting of the fabric.* However, this scale refers to the wetting state of the fabric surface and does not consider the water content that has penetrated into the fabric. Due to the results according to the ISO scale, it is not possible to accurately estimate the level of textile absorption; therefore, dm measurements were additionally performed. Based on the comparison of the ISO scale results with the dm results, these two parameters do not necessarily correlate with each other.

#### 3.2.3. Chemical Liquids Penetration Test

**Figure 15:**
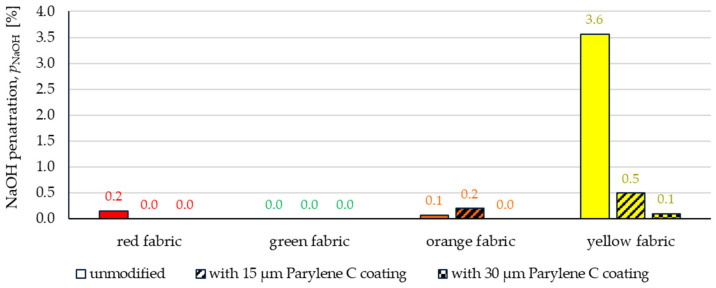
The sodium hydroxide NaOH (30%) penetration through unmodified and coated fabrics.

The results presented in Figure 15, in general, indicate a relatively high resistance of all tested fabrics to penetration by sodium hydroxide (NaOH 30%). Applying the test liquids to the tested fabrics in accordance with the method described in the EN ISO 6530 is a fairly gentle process, similar to splashing a small amount of chemical liquid onto fabric. Thanks to this, for three out of four unmodified fabrics, the indices of penetration (*p*_NaOH_) are practically zero. Only the yellow para-aramid fabric did not show resistance to sodium hydroxide penetration—the value of the *p*_NaOH_ index is 3.6% (Figure 15). However, after the Parylene C coating process, all tested fabrics became resistant to the penetration of sodium hydroxide—the *p*_NaOH_ index values are zero or do not exceed 0.2%.

**Figure 16:**
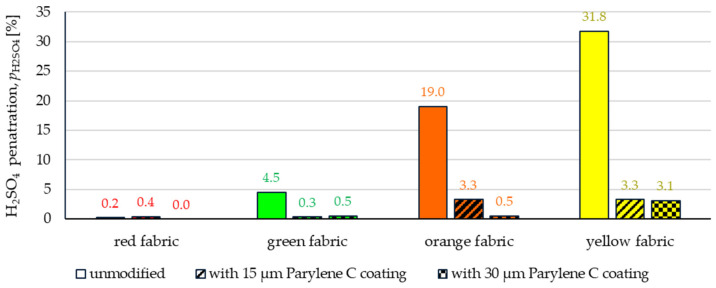
The sulfuric acid H_2_SO_4_ (30%) penetration through unmodified and coated fabrics.

The comparison of Figure 15 and Figure 16 allowed for a conclusion that, in general, the tested fabrics are less resistant to penetration by sulfuric acid than sodium hydroxide. Among the unmodified fabrics, only the waterproof finished red fabric showed considerable resistance to sulfuric acid penetration (value of the *p*_H2SO4_ index is 0.2%), while the *p*_H2SO4_ index values for the remaining fabrics range from 4.5% to 31% (Figure 16).

The method used to modify fabrics with Parylene C resulted in an increase in resistance to sulfuric acid penetration. The *p*_H2SO4_ index values for red and green fabrics are zero or do not exceed 0.5%; however, for orange and yellow fabrics, the values of this index range from 0.5% to 3.3% (Figure 16).

It can be assumed that the reason for the significantly worse results of sulfuric acid penetration tests compared to the results obtained for sodium hydroxide is the significant differences in the surface tension of these liquids. The value of this parameter for sulfuric acid (30%) is 51.7 mN·m^−1^ [31], while for sodium hydroxide (30%)—96.1 mN·m^−1^ [32]. The lower the surface tension of liquid, the greater its ability to penetrate through the fabric. Therefore, sulfuric acid, whose surface tension is approximately two times lower than the surface tension of sodium hydroxide, penetrates the tested fabrics more easily than hydroxide.

#### 3.2.4. Air Permeability

**Figure 17:**
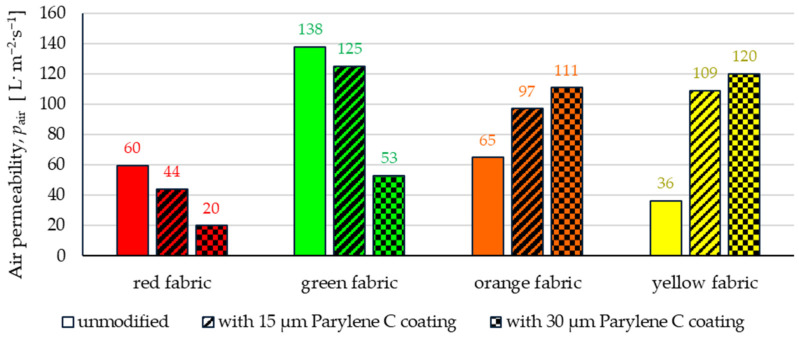
The air permeability of unmodified and coated fabrics.

Figure 17 shows the results of measurements of air permeability of unmodified and coated fabrics.

According to these results, the effect of Parylene deposition had a completely different effect on red and green fabrics than in the case of orange and yellow fabrics. In the case of the first two fabrics, the deposition of an increasingly thick coating of Parylene C resulted in an increasing decrease in air permeability compared to unmodified textiles. In the case of the red fabric, this decrease was 26% for the deposition of 15 μm coating and 66% for the deposition of 30 μm coating. However, in the case of green fabric, this decrease amounted to 9% for the deposition of 15 μm of the coating and 62% for the deposition of 30 μm of the coating. On the other hand, in the case of the other two fabrics, the deposition of an increasingly thick coating of Parylene C resulted in a greater increase in air permeability compared to unmodified textiles. In the case of the orange fabric, this increase was 49% for the deposition of 15 μm coating and 71% for the deposition of 30 μm coating. However, in the case of yellow fabric, this increase amounted to 201% for the deposition of 15 μm of the coating and 231% for the deposition of 30 μm of the coating. The different impact of Parylene C deposition on the air permeability of both pairs of fabrics (red and green, orange and yellow) is probably due to the different impact of the applied coating process on changes in the porosity of the yarn in the abovementioned two pairs of fabrics described in Section 3.1*. The influence of coating on structural properties of fabrics.* Parylene C deposition on red and green fabrics (both with higher weft and warp density, made of staple fibre-based yarns) were less susceptible to yarn compression due to lowering the pressure in the process chamber, compared to the other two fabrics (with lower weft and warp density, made of continuous fibre yarns). Unlike the first two fabrics, in the orange and yellow fabrics, because of yarn compression, wider channels appeared between the weft and warp yarns, thanks to which the air flow resistance is reduced. The presence of a Parylene C coating in the yarn structure (which did not cover these channels) did not have that much importance for reducing air flow. In the case of the red and green fabrics, the deposition of more amount of Parylene C (30 μm) contributed more to reducing the volume of the channels through which the air flowed, because the compression of the twisted and stiff yarn was not as significant as in the case of the other two fabrics.

### 3.3. The Influence of Coating on Electrostatic Properties of Fabrics

#### 3.3.1. Charge Decay

**Figure 18:**
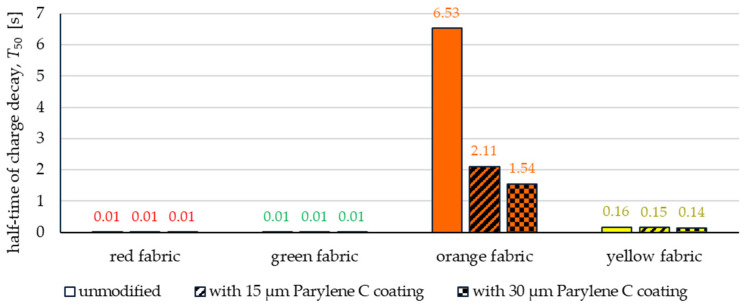
The half-time of charge decay of unmodified and coated fabrics.

Figure 18 shows the results of measurements of half-time of charge decay (*T*_50_) of unmodified and coated fabrics.

According to the results, deposition of Parylene C improved the ability to discharge electric charge only in the case of orange fabric. The deposition of 15 µm coating reduces discharge time by 68%, while the deposition of 30 µm coating reduces discharge time by 76%. The results given in Figure 18 indicate that all fabrics made of aramid fibres (red, green, and yellow) were characterized, both before and after coating with Parylene C, by a very high ability to dissipate the charge. Decay half-time (*T*_50_) for red and green fabrics is below 0.01 s, while for yellow fabrics *T*_50_ values range from 0.14 s to 0.16 s. It should be noted that textiles with a *T*_50_ value of less than 4 s are considered safe and suitable for use in environments where there is a risk of explosion. Considering the above, it is important that the method used to modify the basalt fabric with parylene C resulted in shortening the decay half-time from 6.3 s to below 2 s.

#### 3.3.2. Surface and Vertical Resistance

The results of surface resistance and vertical resistance measurements showed that the deposition of both thinner and thicker Parylene C coating did not change the category of fabrics as electrical insulators. The surface and vertical resistance of unmodified fabrics and those coated with Parylene C was above the range of 10^6^ Ω.

### 3.4. The Influence of Coating on Thermal Insulation Properties of Fabrics

#### 3.4.1. Thermal Conductivity Coefficient

**Figure 19:**
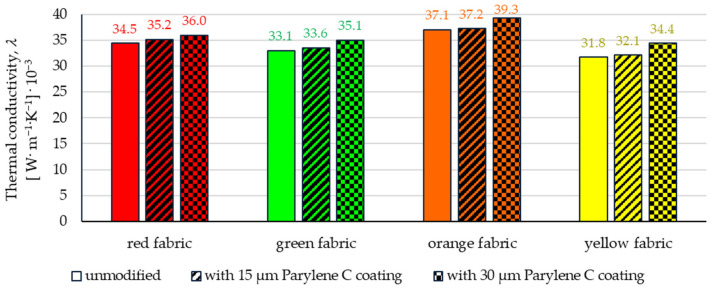
The thermal conductivity of unmodified and coated fabric determined by the Alambeta measurement system.

Figure 19 shows the results of the measurements of the thermal conductivity (*λ*) determined using the Alambeta measurement system. Based on the results, it can be observed that in the case of all fabrics, the increasing content of Parylene C in the fabric causes an increase in *λ*, which in turn reduces their thermal insulation properties.

In the case of the deposition of a 15 µm coating, the highest increase in *λ* compared to unmodified fabrics was observed for red fabric (2.1%), and the lowest for orange fabric (0.4%), while for the deposition of a 30 µm coating, the highest increase in *λ* compared to unmodified fabrics was observed for yellow fabric (8.4%), and the lowest for red fabric (4.5%).

#### 3.4.2. Thermal Imaging

**Figure 20:**
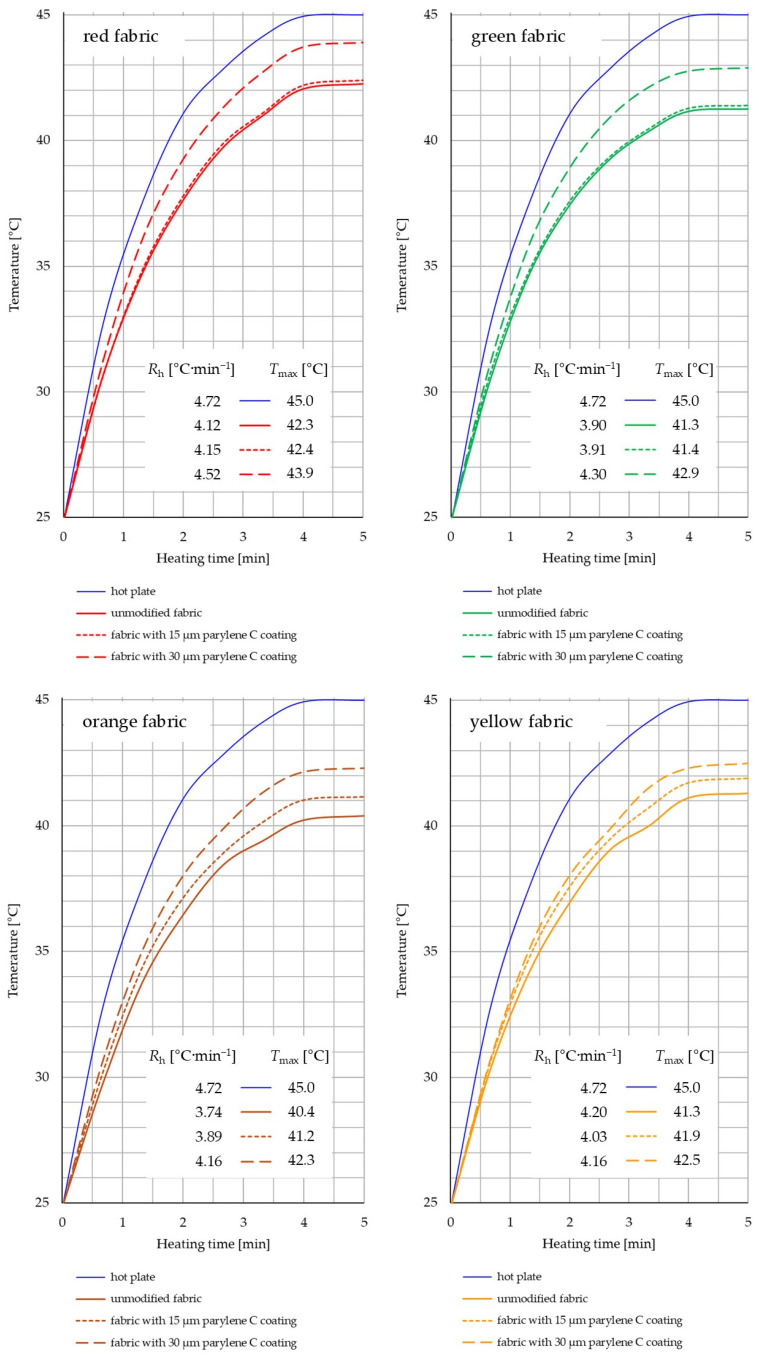
The dependence of the temperature of unmodified and coated fabrics on the heating time of the fabrics on the heat generated by the hot plate.

Figure 20 shows dependence of the temperature of unmodified and coated fabrics on the heating time of the fabrics on the heat generated by the hot plate. In each of the four graphs, where the results for each of the four fabrics are presented separately, the process of heating the hot plate has been marked with a continuous blue line. The hot plate initially has an ambient temperature (25 °C), and after about 4 min, it reaches a maximum temperature *T*_max_ of 45 °C.

Based on the thermal imaging results, it can be observed that they correlate with the results obtained using the Alambeta system. Increasing content of Parylene C in the fabric results in a reduction of the thermal insulation properties of all four tested fabrics, but to a different extent for each of them. Each graph presenting the results for one of the four fabric types contains two parameters describing the curves of fabric temperature increase over time: (1) *R*_h_—the heating rate from the initial temperature (25 °C) to the maximum temperature (reached after approximately 4 min) and (2) *T*_max_—the value of the maximum temperature reached by the fabric. Based on the values of these parameters, it can be observed that in the case of red fabric and green fabric, the deposition of a 15 µm coating caused almost no effect on the thermal insulation of both fabrics. The difference in the *R*_h_ compared to the unmodified textiles is 0.72% (red fabric) and 0.26% (green fabric), while the difference in *T*_max_ compared to the unmodified fabrics is 0.24% (red fabric and green fabric). According to the microtomography results, during the deposition of 15 µm coating on the fabric surface, a continuous layer of Parylene C was not formed because the deposited polymer partially penetrated the free spaces between the fibres of the porous yarn (47% red fabric, 45% green fabric). An additional obstacle for Parylene C monomers on the way to the fabric surface was the fibres protruding above the fabric surface (seen in Figure 2a,b), which could effectively block the flow of Parylene C vapours. These two factors could be the reason why the deposition of a 15 µm layer did not substantially change the porosity of the red and green fabrics, which was confirmed by the results presented in Figure 9 and Figure 10.

A much more notable change in the thermal insulation of the red and green fabrics is the deposition of 30 µm coating. In both fabrics, it causes a significant increase in the *R*_h_ and *T*_max_ values, which makes both modified fabrics significantly less thermally insulating than their unmodified counterparts. The difference in the *R*_h_ compared to the unmodified textiles is 9.71% (red fabric) and 10.26% (green fabric), while the difference in *T*_max_ compared to the unmodified fabrics is 3.78% (red fabric) and 3.87% (green fabric).

The similarity of the results of the effect of the deposition of 15 µm coating on the thermal insulation properties of the red and green fabrics is also influenced by the fact that they were made of the same raw material (meta-aramid staple fibre) and that they differ in thickness by only 2.7%.

In the case of orange and yellow fabrics, the deposition of 15 µm coating causes a significantly greater decrease in the thermal insulation of both fabrics than in the case of red and green fabrics. The difference in the *R*_h_ compared to the unmodified textiles is 4.01% (orange fabric) and 3.87% (yellow fabric), while the difference in *T*_max_ compared to the unmodified fabrics is 1.98% (orange fabric) and 1.45% (yellow fabric). The deposition of 30 µm coating increases this decrease even more, and the difference in the *R*_h_ compared to the unmodified textiles is 11.22% (orange fabric) and 7.99% (yellow fabric), while the difference in *T*_max_ compared to the unmodified fabrics is 4.70% (orange fabric) and 2.91% (yellow fabric). Based on the thermal imaging results, it can also be observed that the unmodified red fabric has the lowest thermal insulation and achieves the highest maximum temperature *T*_max_ = 42.3 °C.

Based on the thermal imaging results, it can also be observed that among the unmodified textiles, the least thermally insulating is the red fabric, which during experiment reaches the highest maximum temperature (*T*_max_ = 42.3 °C), while the most thermally insulating is the orange fabric, which reaches the lowest maximum temperature (*T*_max_ = 40.4 °C). The reason for this is probably the significant differences in spatial geometry and raw material composition between the red fabric and yellow fabrics. The first fabric is clearly thinner than the second one. Moreover, the first has a more compact structure described by the warp and weft density. The more compact yarn’s structure and smaller spaces between twisted yarns in the red fabric (Figure 2) probably resulted in less efficient heat transfer to the colder environment (less efficient cooling) compared to the yellow fabric. The difference weave (twill and plain) could also be significant factor. The influence of the hydrophobic finish in the red fabric cannot be ruled out either.

**Figure 21:**
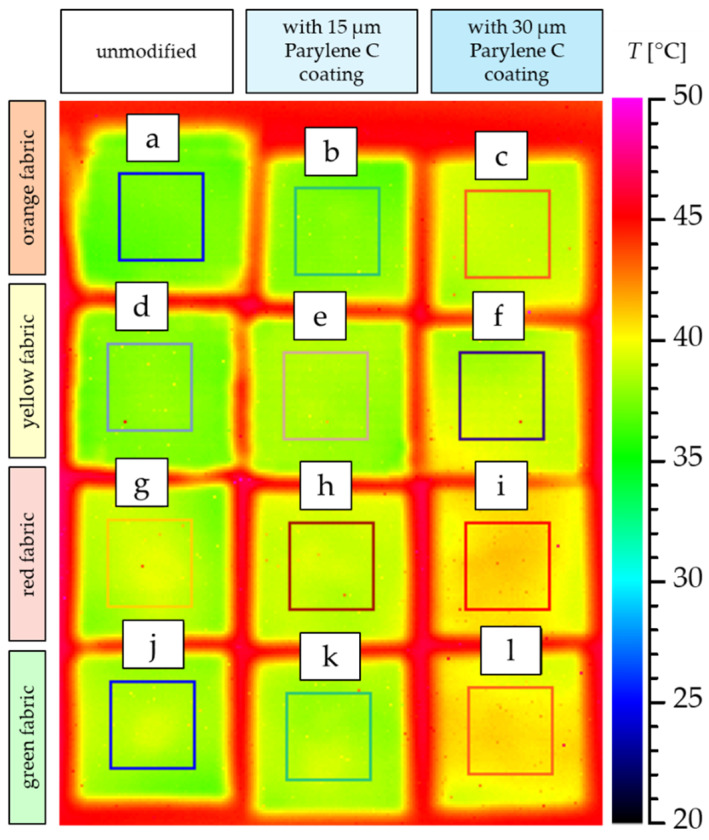
Thermograms illustrating 3rd minute of the heating process of unmodified and coated fabrics placed on a hot plate; orange fabric (**a**–**c**); yellow fabric (**d**–**f**); red fabric (**g**–**i**); green fabric (**j**–**l**).

Figure 21 shows a thermogram of the measurement of the heating process of unmodified and Parylene C-coated fabrics placed on a hot plate.

The thermogram shows the 3rd minute of the process, in which the differences in the temperatures of the upper surface of the tested fabrics resulting from the structural parameters of the textiles, the raw material composition and the presence of the Parylene C coating are clearly visible. The tested samples were in the form of a square with a side of 3 cm. However, in order to reduce the unfavourable effect of the boundary conditions on the accuracy of the measurement, the value of the average temperature of the top surface of the fabrics was taken for a smaller area located in the centre of the sample limited by a square with a side of 2 cm. As can be seen in the thermograms, in the case of all tested fabrics, increasing the content of Parylene C results in increasingly better thermal conductivity of the coated fabrics (the top surface of all four tested fabrics with a 30 µm Parylene C coating reached the highest temperature compared to the fabrics with a thinner coating and unmodified fabrics). The thermograms also confirm the results presented in Figure 20, indicating that the least thermally insulating textiles are the thinnest fabrics: red and green.

### 3.5. The Influence of Coating on Flammability of Fabrics

**Figure 22:**
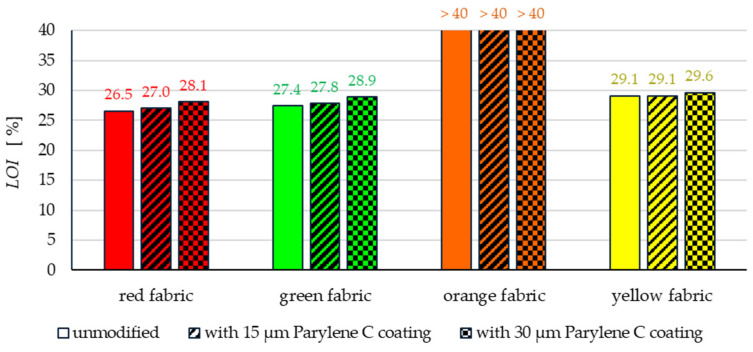
The limiting oxygen index of unmodified and coated fabrics.

The results presented in Figure 22 showed, as expected, a relatively high resistance to ignition for all aramid fabrics, i.e., red, green and yellow fabrics, and confirmed the high fire resistance of basalt fibres from which the orange fabric is made. Among the tested fabrics made of meta-aramid fibres, the red one has a lower *LOI* value (26.5%), compared to 27.4% for the green fabric. The weaker flame-retardant properties of the red fabric may be caused by its waterproof finish. It is known that the presence of some chemical finishing agents, including waterproof ones, e.g., fatty acid resin [33], worsens the flame-retardant properties of textile products.

The main purpose of the flammability tests undertaken was to check whether the fabric modification method used did not negatively affect their flame resistance. The obtained flammability test results should be considered positive. All the aramid fabrics modified with Parylene C showed an increase in the *LOI* value, which means improving their flame retardancy properties. The results given in Figure 22 indicate that this improvement is greater the greater the amount of Parylene C applied to the fabric. In the case of meta-aramid fabrics with the deposition of a 30 μm Parylene C coating, the *LOI* value increases from 26.5% to 28.1% (red fabric) and from 27.4% to 28.9% (green fabric). From a practical point of view, this means a significant improvement in the flame resistance of both these fabrics.

In the case of yellow fabric samples, the *LOI* value increases only slightly from 29.1% for the unmodified fabric to 29.6% for the specimen with the deposition of a 30 μm Parylene C coating. The relatively small improvement in the flammability properties of this fabric after its modification with Parylene C results from the fact that this fabric, even when uncoated, was highly resistant to ignition.

*LOI* values are the basis of some flammability classification systems for various materials, including textiles. The most ignition-resistant category of textile products is defined as “non-combustible” when the *LOI* is above 35%. This category of textiles includes, among others, basalt fibre fabrics, known for excellent heat and fire resistance. When the *LOI* value of a textile material is in the range of 26–35%, this material is called “difficult to ignite” or “self-extinguishing” [34,35]. Textiles falling into this category of flammability include the fabrics made of aramid, modified acrylic fibres and others, which burn in fire but extinguish by themselves when fire is removed.

It should be noted that all tested aramid fabrics, both before and after modification, show *LOI* values above 26%, which means that they meet the criteria of “self-extinguishing” materials.

The flammability tests performed on unmodified and coated orange fabric samples confirmed the previously mentioned outstanding flame resistance of basalt fibres. The fabric samples, both before and after the Parylene C coating process, did not ignite when tested at an oxygen concentration of 40%. Therefore, all tested basalt fabric samples, as expected, can be classified as “non-combustible” materials.

## 4. Conclusions

The article presents research results on the impact of the use of an original, innovative method of deposition of Parylene C on the functional properties of textiles with potential applications in various fields, such as thermal protective clothing, chemical protective clothing, protective footwear, packaging, barrier materials, etc. The evaluation of the effects of the method used was based on interdisciplinary studies considering the impact of fabric coating on changes in their: structure, surface properties, barrier properties protecting against the harmful effects of liquid chemicals, electrostatic properties, properties regulating heat and mass transfer through them and flammability properties. Based on the results obtained, the following conclusions can be drawn:

- According to the results of micro-CT analysis, the Parylene C deposition method used is a controlled process in terms of coating thickness on textile substrates. Despite the complicated geometric structure of textile substrates, the results confirmed that both assumed average thicknesses of Parylene C coatings on fabrics (15 μm and 30 μm) were similar to the actual ones. Larger differences between the assumed and actual average thickness of the Parylene C coating were observed in the case of deposition of the thinner coating.
- Micro-CT 3D visualizations of fabrics (Figure 11) showed that in the case of deposition of a 15 μm Parylene C coating on the fabric surface, the coating is discontinuous because a significant part of Parylene C penetrates the fabrics’ structure, especially those characterized by higher porosity (Figure 9). Deposition of a thicker coating of Parylene C (30 μm) results in the formation of a more extensive and continuous layer on the surface of fabrics, especially those made of filament yarns.
- Wetting tests showed that the deposition of even a thinner coating of Parylene C (15 μm) gives the fabrics’ surface a hydrophobic character (contact angle values range from 124° to 139°). The highest contact angle (139°) was found for the red fabric, which is due to the fact that it was hydrophobic-finished before modification with Parylene C. The spray test results (Figure 14) showed that with the exception of this red hydrophobic-finished fabric, the deposited Parylene C coatings cause significantly lower water absorption by fabrics compared to unmodified fabrics (water absorption is lower by 71% to 78% depending on the fabric). Generally speaking, applied deposition of Parylene C is an effective method of hydrophobizing the fabrics’ surfaces, including fabrics with a high water wettability. The results of chemical liquids penetration through fabrics correlate with the results of the spray test. Deposited coatings of Parylene C cause much less acid penetration through fabrics compared to unmodified fabrics (H_2_SO_4_ penetration is lower from 83% to 97% depending on the fabric). This confirms the high chemical resistance of Parylene C applied to fabrics.
- Depending on the type of yarn the fabrics were made of (continuous or staple fibres and more or less porous), the Parylene C deposition had a different effect on the air permeability of the textiles (Figure 17). In the case of fabrics made of continuous fibres (orange and yellow), the factor determining the increase in air permeability of modified fabrics was the phenomenon of yarn compression, which increased the air flow through the fabrics (from 49% to 231% depending on the fabric and coating thickness). In the case of fabrics made of staple fibres (red and green), the twisted and more stiff yarns did not undergo compression during modification with Parylene C. The main factor determining the decrease in air permeability of these fabrics due to their modification was the deposition of Parylene C in the yarns’ structure, reducing free spaces between the fibres, increasing flow resistance and ultimately reducing their air permeability (from 9% to 66% depending on the fabric and coating thickness).
- Deposited coatings of Parylene C significantly influenced the electrostatic properties of only the orange fabric (made of basalt fibres), causing the decay half-time of the electric charge to drop below 4 s (*T*_50_ < 4 s qualifies the products for use in areas where there is a risk of explosion). The remaining three fabrics in the unmodified version and those with a Parylene C coating meet this safety criterion. The results of surface and vertical resistance do not change the category of fabrics as electrical insulators (both resistances for unmodified fabrics and fabrics with a Parylene C coating reached values above 10^6^ Ω).
- Based on the results of thermal insulation properties tests, fabrics coated with Parylene C are characterized by a higher thermal conductivity coefficient than unmodified fabrics (the coefficient increases with the increase in coating thickness). These results were confirmed by thermal imaging, according to which, in particular, the deposition of a thicker coating of Parylene C (30 μm) contributes to a significantly greater increase in the fabric heating rate *R*_h_ compared to unmodified fabrics.
- The flammability test results showed that in the case of all aramids fibre fabrics, the Parylene C deposition method increases the *LOI* value of these fabrics, which means improving their flame retardancy properties. Samples of unmodified basalt fabric characterized by exceptionally high flame resistance retained this feature also after coating with Parylene C and can be classified as non-combustible materials.

## Figures and Tables

**Table 1:** The physical parameters of the tested fabrics.

Fabric Name	Composition	Thickness ^(a)^ [mm]	Mass per Unit Area ^(b)^ [g·m^−2^]	Weave	Warp Density [mm^−1^]	Weft Density [mm^−1^]	Total Porosity ^(c)^ [%]	Yarn Porosity ^(c)^ [%]
red	hydrophobic-finished meta-aramid	0.37	233	Twill	3.01	2.25	66	47
green	meta-aramid	0.36	175	Plain	2.57	2.74	67	45
orange	basalt	0.50	380	Plain	0.82	0.89	45	11
yellow	para-aramid	0.45	214	Plain	0.62	0.64	66	58

^(a)^ determined according to PN-EN ISO 5084:1999 [17]; ^(b)^ determined according to PN EN 12127:2000 [18]; ^(c)^ determined by X-ray micro-CT.

## Data Availability

The original contributions presented in the study are included in the article, further inquiries can be directed to the corresponding authors.
